# Thalidomide upper limb embryopathy – pathogenesis, past and present management and future considerations

**DOI:** 10.1177/17531934231177425

**Published:** 2023-05-24

**Authors:** Neil Vargesson, Geoffrey Hooper, Grey Giddins, Alastair Hunter, Paul Stirling, Wee Lam

**Affiliations:** 1 School of Medicine Medical Sciences & Nutrition. Institute of Medical Sciences. University of Aberdeen. Aberdeen, UK; 2 Princess Margaret Rose Orthopaedic Hospital, Edinburgh, UK; 3 Royal United Hospitals, Bath, UK; 4 Department of Trauma and Orthopaedics, University College London Hospitals, London, UK; 5 Fife Hand Clinic, Queen Margaret Hospital, Dunfermline, UK; 6 Royal Hospital for Children and Young People, Edinburgh, UK

**Keywords:** Thalidomide, radial dysplasia, reversed shoulder, embryopathy, cereblon

## Abstract

This review article provides a comprehensive overview of thalidomide upper limb embryopathy including updates about its pathogenesis, a historical account of the management of the paediatric thalidomide patient, experience with management of the adult patient, as well as creating awareness about early onset age-related changes associated with limb differences. Despite its withdrawal from the market in November 1961, novel discoveries have meant thalidomide is licensed again and currently still in use to treat a variety of conditions, including inflammatory disorders and some cancers. Yet, if not used safely, thalidomide still has the potential to cause damage to the embryo. Recent work identifying thalidomide analogues that retain clinical benefits yet without the harmful effects are showing great promise. Understanding the problems thalidomide survivors face as they age can allow surgeons to support their unique healthcare issues and translate these principles of care to other congenital upper limb differences.

## Introduction

Thalidomide was marketed in several countries worldwide as a sedative between 1957 and 1962, and rapidly gained popularity as an effective drug for relieving morning sickness in expectant mothers. No screening was undertaken in humans prior to approval by drug licensing authorities, although drug screening in animal models demonstrated apparent drug safety. In the early 1960s, it was suggested that an increase in the number of congenital abnormalities may be linked to maternal ingestion of thalidomide (Lenz, 1962; McBride, 1961). The evidence continued to grow and by late November 1961, when thalidomide was withdrawn from the market, the teratogenic effects of thalidomide were indisputable (Vargesson, 2019; Vargesson and Stephens, 2021).

One of the most striking teratogenic effects of thalidomide was seen in the upper limbs, with congenital upper limb differences occurring in up to 87% of thalidomide survivors (Mansour et al., 2019). These ranged from milder conditions like Blauth II or III thumb hypoplasia, to Bayne IV radial dysplasia with complete thumb absence and in the most severe cases, phocomelia and amelia. Upper limb thalidomide embryopathy was typically bilateral and more severe in the left than right upper limb, for reasons unknown. In addition to the upper limbs, most other tissues and organs in the body could be affected, leading to lower limb abnormalities, hearing problems, micro-ophthalmia, spinal issues, internal organ damage or damage to the cardiovascular, renal and gastrointestinal systems (Mansour et al., 2019; Vargesson, 2019).

Many lessons have been learned from this tragedy, which affected thousands of newborns, in the late 1950s and early 1960s, and their families. Many of those who survived were treated surgically as children. These survivors are now aged between 59 and 64 years, and some are experiencing early onset age-related changes in adjacent joints with further adverse effects on their quality of life (Markiewicz et al., 2023).

In this article, we provide an update on the pathogenesis of thalidomide upper limb embryopathy and a historical account of upper limb management in affected patients. We also reassess the present management of patients, especially as they reach their seventh decade of life with age-related problems. It is hoped that this article will provide some important pointers for the future management of these patients and also allow us to revisit lessons learned from this tragedy.

## Update of the pathogenesis and mechanism of thalidomide embryopathy

Thalidomide has numerous actions on the body. It has anti-inflammatory, immunomodulatory, anti-angiogenic and anti-myeloma actions as well as actions on the nervous system, where it can be neuroprotective as well as cause peripheral neuropathy after long-term exposure (Vargesson, 2019). Given these actions, thalidomide and its sister analogues lenalidomide and pomalidomide, are now used as effective treatments for several conditions, including multiple myeloma and the complications of leprosy (Vargesson, 2019). Thalidomide has also been shown to effectively treat vascular disorders, including hereditary haemorrhagic telengiectasia (HHT) (Lebrin et al., 2010; Peng et al., 2015), small bowel vascular malformations (Tang et al., 2020) and recently, radiation induced brain inflammation (Cheng et al., 2023). Thalidomide is known to be broken down into active metabolites either by spontaneous hydrolysis in body fluids or through the cytochrome P450 enzyme system in the liver (Franks et al., 2004; Vargesson, 2015). With thalidomide in clinical use and its teratogenic effects still present, great care must be taken when administering the drug to patients; patient protection schemes are in place in many areas of the world to ensure it is taken and used safely (Mueller and Lewis, 2021). The need for such schemes is emphasized by recent reports of cases of thalidomide embryopathy in Brazil, where thalidomide is used to treat endemic leprosy (Vianna et al., 2011).

It is well known that thalidomide causes damage to the upper limbs and other external tissues of the body (i.e. face, ears, eyes, genitals, etc.) in a short time sensitive window in the first trimester of human development. How does thalidomide cause birth differences? Recent research indicates that there are three favoured models of how thalidomide causes damage to the forming embryo, however these are unlikely to be mutually exclusive.

### Cereblon

Cereblon (CRBN) is a ubiquitin ligase whose role is to tag other molecules for destruction when they are no longer required. Cereblon is bound by thalidomide to form a complex; recent experiments demonstrated that when exposed to engineered versions of cereblon that cannot bind thalidomide, zebrafish and chicken embryos remained unaffected by the modified version of thalidomide (Ito et al., 2010). Since this finding, the cereblon–thalidomide complex has been found to bind to and modulate the expression of other genes, including Ikaros and Aiolos, which are involved in the anti-myeloma action of thalidomide but also in repressing the expression of molecules, such as SALL4, p63 and PLZF, among others that have important functions in limb development (Asatsuma-Okumura et al., 2019, 2020; Donovan et al., 2018; Matyskiela et al., 2018; Yamanaka et al., 2021). Moreover, in humans who possess SALL4 genetic variations, this leads to Duane-radial ray syndrome, in which there are limb and internal organ differences that can look remarkably similar to thalidomide embryopathy, and which has been confused with this condition (Kohlhase et al., 2004, 2005). To date, it has not been conclusively demonstrated that SALL4 is responsible for all the damage seen in thalidomide survivors and birth differences between thalidomide embryopathy and Duane-radial ray syndrome do differ. Nonetheless, the fact that multiple different genes have now been linked to the cereblon–thalidomide complex indicates that thalidomide is likely to influence multiple gene targets and pathways in timing-dependent ways (Asatsuma-Okumura et al., 2020; Vargesson, 2019).

### Induction of cell death and reactive oxygen species

Thalidomide can induce cell death in embryonic tissues and also prevent cell proliferation (Knobloch et al., 2007, 2011). It has also been shown to induce reactive oxygen species, which themselves can lead to cell death causing tissue damage. Moreover, preventing embryos from producing reactive oxygen species makes the embryos resistant to thalidomide embryopathy (Parman et al., 1999).

### Inhibitory action on the blood vessels

In chicken embryos the anti-angiogenic action of thalidomide has been shown to cause rapid blood vessel loss, induce cell death and cause a range of limb differences that look remarkably similar to those in thalidomide-exposed humans (Davey et al., 2018; Therapontos et al., 2009). The other known actions of thalidomide, namely anti-inflammatory and neurotoxic actions, do not seem to harm the development of the chicken embryo (Mahony et al., 2018; Therapontos et al., 2009).

Ongoing work in this area has identified many vascular specific molecular targets that are influenced by thalidomide (Ema et al., 2010; Vargesson, 2015; Yabu et al., 2005). Vasoprotective agents such as nitric oxide can prevent thalidomide-induced antiangiogenic actions (Siamwala et al., 2012; Tamilarasan et al., 2006). Importantly, thalidomide seems to target newly formed or developing blood vessels that do not possess a protective vascular smooth muscle coat, whereas those that possess a smooth muscle coat are unharmed and develop normally (Therapontos et al., 2009; Vargesson and Hootnick, 2017). In early limb and embryonic development, there are more smooth muscle negative vessels present which are susceptible to damage by thalidomide, while later in limb and embryonic development blood vessels are mature with smooth muscle coats and therefore thalidomide damage is much less severe (Therapontos et al., 2009; Vargesson, 2015, 2019). Thus, the range of limb differences (and other embryonic damage) induced by thalidomide could be attributed to the timing of exposure and blood vessel maturity.

Notwithstanding that limb development outgrowth and patterning is controlled by important molecules, such as Fibroblast Growth Factor 8 (Fgf8) and Sonic Hedgehog (Shh), respectively, and that both these molecules have been shown to be downregulated by thalidomide in chicken, zebrafish and rabbit studies, it remains unclear if this action is direct or indirect (for example through vascular inhibition and tissue loss) (Hansen et al., 2002a, 2002b; Ito et al., 2010; Knobloch et al., 2011; Therapontos et al., 2009).

The longer-term effects of vascular damage remain areas of current research interest. For example, it can be difficult in some thalidomide patients to palpate a pulse in the arms and take blood pressure readings as the vascular patterns are altered (Tajima et al., 2016). Mechanistically this is likely, either, (i) because the vessels did not form and are not there or are in different locations, with the resulting tissue damage causing further changes to vascular patterning events and further exacerbating the damage. Or (ii) the vascular abnormalities and changes could be secondary to loss of molecules like SALL4, p63, PLZF (and others) causing loss of cells or failure of cells to proliferate, migrate and differentiate into the correct tissues in the correct places. The resulting limb damage would then be exacerbated by loss or misplacement of blood vessels and later in development, by nerve innervation being altered as a result of the tissue loss. Alternatively (and more likely in the opinion of the Authors) it may be caused by both of the above mechanisms, and which is dependent on the timing of limb/embryo development and exposure to thalidomide.

### Recent updates

Recent work indicates that the anti-angiogenic action of thalidomide can also occur in a cereblon-independent manner, highlighting that the action of thalidomide on the blood vessels could be direct as well as through other molecular targets (Beedie et al., 2020; Heim et al., 2021; Peach et al., 2020). Indeed, recent research indicates that cereblon, p63, SALL4, EGFL6 are expressed in, or have roles in, the development of blood vessels, suggesting thalidomide could influence these molecules in developing vessels (Beedie et al., 2020; Chandrangsu and Sappayatosok, 2016; Tang et al., 2020). Whether these molecules also underpin the anti-angiogenic action of thalidomide directly remains unknown.

Furthermore, it has been known for a long time that thalidomide is broken down into active metabolites in two ways, either through the cytochrome P450 (CYP2C19 and CYP3A) enzyme pathway in the liver, or by hydrolysis in body fluids (Chowdhury et al., 2010; Franks et al., 2004; Yamazaki et al., 2012). Previous work has indicated that some of these active metabolites could be causing some of the actions of thalidomide; for example, metabolic activation seems to be required for the drugs anti-angiogenic action and its teratogenic action (Bauer et al., 1998; Franks et al., 2004; Vargesson, 2013). Indeed, recent work has shown that the damage caused by thalidomide in zebrafish embryos is more pronounced in those expressing human cytochrome P450 enzymes (CYP3A), indicating that the cytochrome P450 pathway (which breaks down thalidomide) may be responsible for the teratogenic actions of thalidomide in humans (Dong et al., 2023). This has been further demonstrated by recent work showing a major metabolite of thalidomide, 5-hydroxythalidomide, was identified to bind cereblon and form a CRBN-SALL4 complex leading to SALL4 degradation, which has been linked with some of the teratogenic actions of thalidomide (see earlier section). While thalidomide itself induced the formation of a CRBN-Ikaros complex only; with Ikaros underpinning the drug’s immunomodulatory actions (Furihata et al., 2020). These findings would suggest the ability of thalidomide and its metabolites to form CRBN complexes with different molecules, may underpin some or many of the different actions/functions of thalidomide. Yet, as discussed, thalidomide’s antiangiogenic action can also harm the embryo and also occur in a cereblon independent manner (Beedie et al., 2020; Heim et al., 2021; Peach et al., 2020). When taken altogether, this then might explain the pleiotropic and variable nature of the damage seen between survivors through thalidomide potentially utilizing multiple mechanisms of action in the embryo. Indeed, we know thalidomide has many clinically relevant actions in the adult, including anti-inflammatory, anti-myeloma, anti-angiogenic as well as neuroprotective actions, and which would likely be devastating in the developing embryo.

In summary, while great progress has been made in the last few years in our understanding of the mechanism underlying thalidomide upper limb embryopathy, the precise mechanism remains to be determined. It is likely that the cereblon pathway, the ability of the drug to induce cell death, inhibit cell proliferation and its antiangiogenic actions, are all involved. The search for the exact mechanisms continues and remains as important as ever as we see ongoing or new medical issues in thalidomide patients who are experiencing early onset age-related changes, such as neuropathy (Markiewicz et al., 2023). The rest of the article examines some of the past and present clinical management of these patients.

## Early management of the paediatric patient with a focus on surgery and prostheses

Many of the thalidomide-affected infants who survived in the 1950s and 1960s had upper limb anomalies of various types but tending to be of the most severe kind. Absence of the upper limb, vestigial digits attached to the shoulder, flipper-like limbs and various forms of radial dysplasia were all seen. Most children had both their upper limbs affected in a similar pattern. In addition, many with upper limb anomalies also had very severe lower limb deficits, and problems with sight and hearing as well as gut and cardiac anomalies. The management of the upper limb problems could be seriously affected by these associated conditions.

There were few centres in the 1960s and 1970s that had the expertise to deal with the onslaught from these severe congenital problems, which had previously been rare. In Scotland, the Princess Margaret Rose Orthopaedic Hospital (now closed) in Edinburgh became a national referral centre because the necessary surgical expertise was available. A bioengineering centre was established for the development and fitting of prosthetic limbs and appliances, both for upper and lower limb problems. A self-care unit allowed the children and their parents to stay in the hospital while being assessed by surgeons, prosthetists, occupational therapists and physiotherapists; this was important because many of the children had come from a long distance away, and time was required to assess their needs. The multidisciplinary approach was essential.

**Figure 1.**
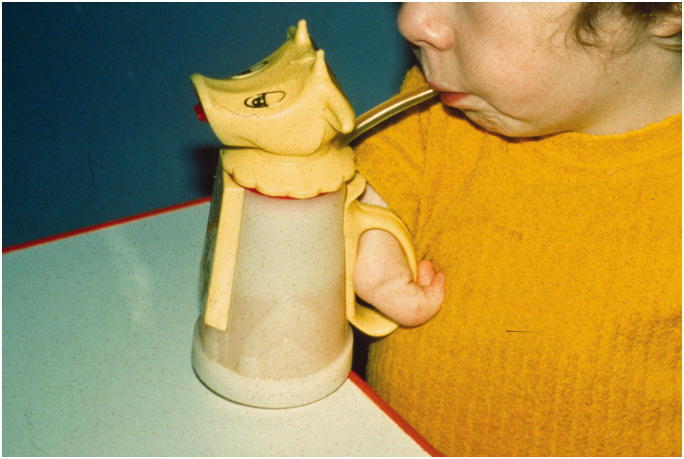
Simple orthoses could help with daily activities.

The therapists had an important role in teaching the children to use simple adaptations and appliances for self-care activities, such as dressing and perineal hygiene, and to use the mouth for holding and manipulating objects (Figure 1). Initially it was thought that powered prostheses would help in the management of upper limb problems in children affected by thalidomide, but the upper limb prostheses then available, either cord operated or gas powered, had almost no role to play in improving upper limb function (Nichols et al., 1968). They were too cumbersome and children who had relatively good function in the lower limbs rapidly learned to use their legs and feet for functions normally caried out with the arms and hands, usually with great dexterity.

Clearly no surgical treatment was appropriate for a missing limb, or a vestigial digit attached to the trunk, although the latter structure might be used to operate a switch on a powered prosthesis. There was a small place for passive, cosmetic prostheses to fill the clothes of those with completely missing upper limbs.

**Figure 2.**
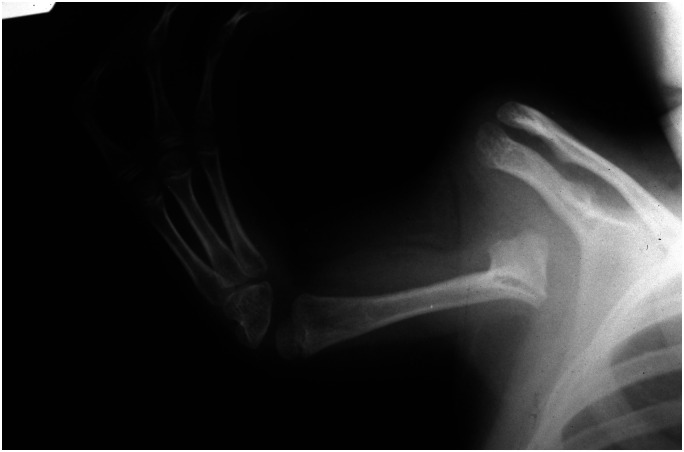
A typical example of thalidomide-associated ‘phocomelia’. Note the presence of the scapula and clavicle. The hand has a major radial deficit and is attached to a short bone that has some features of a humerus.

In addition, the nomenclature for congenital hand differences was very much in development during the thalidomide era. The term ‘phocomelia’ was commonly applied to any anomaly in which a recognizable but highly abnormal hand was attached to the shoulder region. The clavicle was present and usually normal, and there was a scapula, but the shoulder joint itself was always absent, meaning that the hand was not stable on the trunk (Lamb et al., 1971) (Figure 2). Re-examination of radiographs from these ‘phocomelic’ upper limbs has shown that almost none of them had the standard types of intercalary deficits described by Frantz and O’Rahilly (1961) before the thalidomide tragedy; most were extreme forms of longitudinal defects (Goldfarb et al., 2005; Tytherleigh-Strong and Hooper, 2003). These short and unstable upper limbs were of little functional use, especially if they were too short to come together within the child’s visual field; such grasping movements using both upper limbs are one of the first purposeful ones made by an infant. Attempts were made to overcome this problem surgically by combining stabilization of the hand with lengthening the upper limb. This was done by mobilizing the clavicle from its sternal attachment and turning it down to attach it to the hand, producing a rudimentary shoulder and with some potential for growth in the upper limb (Sulamaa and Ryöppy, 1964). Although the originators reported encouraging early results, the procedure did not come into wider use and the non-surgical alternatives of therapeutic training were probably more effective.

Where surgery did have a major role was in the treatment of the various forms of radial dysplasia, one of the characteristic types of upper limb anomaly in those affected by thalidomide. Much experience was gained in the management of radially deviated hands, by centralization (Lamb, 1977) and radialization (Buck-Gramcko, 1985) of the hand on the wrist, and in the treatment of absent thumbs by pollicization (Buck-Gramcko, 1971). Although doubt has been cast on the utility of wrist realignment procedures (Ezaki, 2021), a longer-term follow-up of adult patients treated by centralization (most with added pollicizations) in childhood found that their function was generally good (Lamb et al., 1997). However, the forearms were short and there was usually some recurrence of radial deviation. Other less common upper limb anomalies associated with thalidomide were treated on an individual basis.

The thalidomide tragedy brought undue suffering, but the management of these children via a one-stop multidisciplinary centre allowed some hope and respite to many children and their families. The longer-term study (Lamb et al., 1997) also found that many children grew up to be well-adjusted adults who had married in their 20s and 30s and had children of their own, and most were gainfully employed. Nevertheless, the longer-term effects of thalidomide on the upper limb remains unknown as these adults reach their seventh decade of life.

## Age-related problems in upper limb thalidomide embryopathy

**Figure 3.**
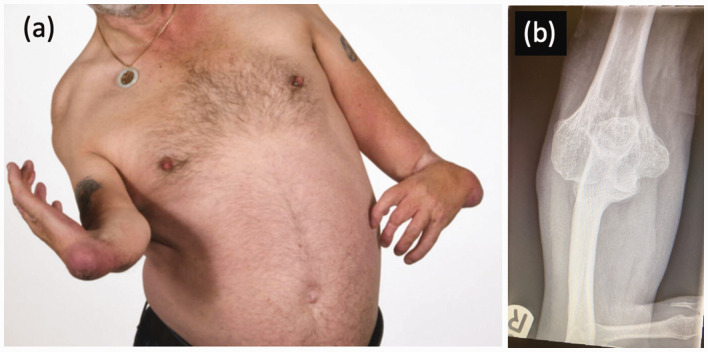
(a) An adult thalidomide patient with bilateral radial dysplasia. (b) The right elbow joint, demonstrating a single bone articulation that can be compensated for by glenohumeral external rotation.

**Figure 4.**
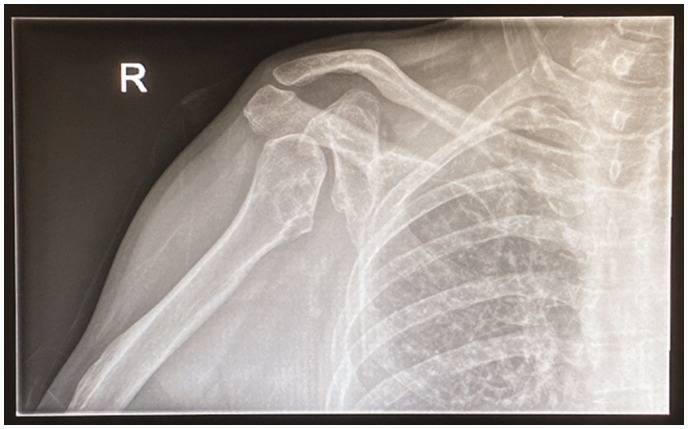
Radiological changes of the right shoulder joint showing severe glenoid dysplasia, with concave remodelling of the humeral head. This is due to constant excessive external rotation at the shoulder and has been described as a ‘natural reverse shoulder replacement’.

Ruffing (1977) reported on a series of children with thalidomide embryopathy and introduced the concept of ‘hidden defects’, differences that were not immediately obvious on clinical presentation that may only become apparent with the passage of time. It was specifically noted that ‘early osteoarthritis is expected to occur in the future’. For example, with a missing radius, forearm rotation is affected, and the elbow is often stiff (Figure 3). The younger patient can compensate by constant excessive external rotation at the shoulder, but over time this may result in damage to the glenohumeral joint, leading to glenoid dysplasia and concave remodelling of the humeral head. The overall effect of this remodelling is to move the centre of rotation of the glenohumeral joint inferiorly and medially, optimizing the deltoid muscle lever arm for improved glenohumeral stability and function. These are the same principles that are harnessed in reverse geometry shoulder arthroplasty, and this pattern of change has been described as development of a ‘natural’ reverse shoulder replacement (Kimmeyer et al., 2021) (Figure 4).

The incidence of such ‘hidden’ age-related changes in these patients may be underestimated. The approximately 450 thalidomide survivors in the UK are now aged between 59 and 64, a time of onset of symptomatic degenerative musculoskeletal pathology, even for a population of this age without congenital differences. The emergence of hidden defects, combined with the cumulative effects of altered joint biomechanics with increasing age may predispose survivors to such pathology. Ghassemi Jahani et al. (2014) and Newbronner et al. (2019) found that the prevalence of arthritis is increased in thalidomide survivors as compared with the general population, affecting around 93% of patients at a mean age of 46 years. Even in their fourth decade, these survivors already showed worse patient-reported outcome measures (PROMs) as compared with baseline population values (Ghassemi Jahani et al., 2014). To our knowledge, there are no specific longitudinal studies with paired data, but comparison of PROMs between patients with a mean age of 46 in one study (Ghassemi Jahani et al., 2014) and a mean age of over 60 in another study (Markiewicz et al., 2023) revealed significantly worse function in older survivors.

The health-related quality of life (HRQoL) of survivors is also significantly worse than baseline population values. Despite increasing awareness and publication of the functional and HRQoL problems faced by survivors, up to 43% of survivors report difficulty accessing healthcare services (Nippert et al., 2002), with survey data showing that survivors perceive a lack of understanding of complex care needs as a significant barrier.

The hand surgery community must be mindful of the physical and social challenges faced by this group of patients to ensure ongoing appropriate care in the context of deteriorating disability. Managing the upper limb problems in this unique adult patient population will require easier and more direct access to appropriate surgical expertise. It is also important that hand surgery colleagues share their experience: although the number of referrals of these adult patients is likely to be small, it will probably increase as they age and access to care becomes more widely available.

## Management of the adult patient

As with any complex upper limb disorder, a holistic approach is emphasized in the management of the adult patient with thalidomide embryopathy. Working with multidisciplinary specialists with experience of these adult patients, including neurologists, dermatologists and therapists, is important. National organizations with extensive networks, such as The Thalidomide Trust in the UK, can provide additional support for patients and clinicians.

The most common presentations are pain, increasing restriction with activities of daily living and nerve-related symptoms, particularly carpal tunnel syndrome (Ohnishi and Hinoshita, 2017). The assessment focuses on the history of the presenting symptoms while taking into account how this affects their broader function, as pain and decreasing joint movement owing to new onset adult symptoms compound existing impairments (Newbronner and Atkin, 2018). It is important to gain a detailed understanding of their level of independence, activities of daily living and support network in order to inform what treatment options might be practical and reliable. A surgical history will be especially relevant as most adults with thalidomide embryopathy will have had some surgery previously, the majority as children (Ghassemi Jahani et al., 2017). They will also present with the standard co-morbidities of any adult of their age, so a thorough past medical and drug history will be essential in assessing the surgical and anaesthetic risks.

Clinical examination should encompass the entire upper limb, while taking into account any lower limb pathology. The pattern of upper limb difference is typically unique to that individual; as discussed, the commonest ones include limb shortening, radial longitudinal deficiency and asymmetry. Up-to-date nomenclature and classification of these differences should probably be used for communication with other congenital hand specialists and also to gather meaningful data, going forward (Chan et al., 2023).

The active and passive ranges of movement should be measured and recorded, and a detailed neurological examination is required especially in the presence of any evolving neurological symptoms. Generalized alterations in sensation are common in patients with thalidomide embryopathy (Nicotra et al., 2016) and may include cervical spinal nerve entrapment. A functional assessment with a physiotherapist or occupational therapist is often valuable in gaining a broader perspective on functional status and on how to optimize upper limb symptoms, especially when considering more complex upper limb surgery. The use of PROMs or other health-related questionnaires should become an established part of assessment in these patients. Radiographs should include the affected joint and possibly the entire upper limb, to allow a clear picture of the bony anatomy and joint pathology. Gaining orthogonal views of each joint may prove difficult owing to fixed flexion deformities. Computed tomography (CT) scans are particularly helpful when assessing the patterns of deformity and planning surgery.

**Figure 5.**
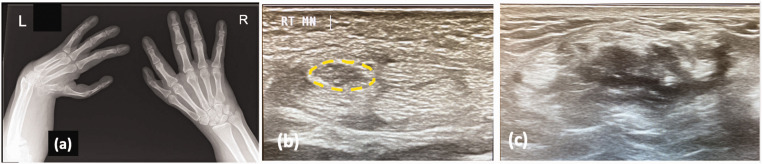
(a) This patient with bilateral radial dysplasia presented with severe median nerve compression in his right hand. Note the scaphoid hypoplasia on the right wrist indicating a mild radial dysplasia with thumb hypoplasia. (b) Ultrasound images showed an enlarged median nerve proximal to the carpal tunnel (yellow dotted line). Such imaging is helpful when planning surgery as the position of the nerve may be abnormal and (c) The left wrist was also scanned but no median nerve could be seen convincingly.

Nerve-related symptoms are one of the commonest presentations in adult thalidomide patients. Carpal tunnel decompression (CTD) is the most common upper limb procedure in adult patients with thalidomide embryopathy (Ghassemi Jahani et al., 2017). Deformity arising from radial longitudinal deficiency, for example extreme wrist flexion may lead to an increased risk of median nerve compression at the wrist (Figure 5). Various studies suggest the drug can also cause a direct axonal injury that is dose-dependent (Zara et al., 2008). Reports of patients who developed late-onset neuropathy after ingesting thalidomide as adults would also suggest there is a direct neurotoxicity. Rose et al. (2005) reported two adult patients, one treated with thalidomide for myeloma and the other for Behçet disease who developed lower limb neuropathy and were successfully treated with decompression operations. The proposed mechanisms include inhibition of TNF-alpha and NF-Kappa-B, which are required for sensory nerve survival (Mileshkin and Prince, 2006; Umapathi and Chaudhry, 2005); in addition, capillary damage from long-term exposure to thalidomide resulting in axonal cell death has also been proposed (Grammatico et al., 2016). Further research in this area should yield important insights in the pathogenesis of this common side effect.

In patients with peripheral nerve-related symptoms, nerve conduction studies should be done, ideally by a neurophysiologist with experience of adult thalidomide patients. A mixed picture of nerve compression and polyneuropathy is often found, but if there is a congenital difference with altered wrist anatomy this can mean the typical electrophysiological changes in carpal tunnel syndrome are unhelpful (Nicotra et al., 2016). Ultrasound scanning can aid in surgical planning for nerve release in determining any variation in the anatomy and path of the nerve. The published data indicate that CTD improves the sensory symptoms reliably (Oshima et al., 2006) but consideration should be given to performing an extended median nerve release at the wrist, as the normal anatomical landmarks and path of the nerve may be unreliable.

Pain at the wrist due to dysplasia and repetitive overloading can also result in osteoarthritis. After exhausting non-operative treatment options, the choices for salvage procedures are denervation, partial wrist arthrodesis or a proximal row carpectomy, if feasible, and total wrist arthrodesis. Great care needs to be taken in positioning a total wrist arthrodesis; the previous ‘abnormal’ wrist posture may be essential for a patient with thalidomide embryopathy to perform some daily tasks. The abnormal anatomy and smaller bones make the operation technically more difficult and may require use of non-standard implants. There is currently no role for wrist arthroplasty given the likely longstanding wrist imbalance.

As noted above, patients with skeletal dysplasia are more likely to develop degenerative shoulder disease at a younger age than the general population (Sewell et al., 2014). Non-operative management includes physiotherapy and joint injections and forms the mainstay of treatment. Shoulder arthroplasty in this group of patients is very rarely done. There are a few reported cases recorded, but robust long-term data are not available (Merkle et al., 2016; Newman, 1999;  Sewell et al., 2014,). Options include resurfacing hemiarthroplasty or total shoulder arthroplasty with some evidence of improvement in pain and function, but more complications are to be expected than in the general population. In this context, great caution must be exercised when considering treatment options for shoulder pain. The authors have seen cases of shoulder arthroplasty requiring early revision with poor outcomes worse than before surgery. For this reason, we consider that any major joint surgery in the upper limb (arthroplasty or arthrodesis) should only be done after a detailed multi-disciplinary team discussion, with substantial warnings to the patient. The authors are aware of one case of elbow arthroplasty in a patient with thalidomide embryopathy but with a poor result.

Early preoperative assessment with an experienced anaesthetist can help to reduce delay to surgery or cancellations. On the day of surgery, venepuncture can be very challenging owing to shortening limbs and abnormal vasculature, as discussed above. Intubation may be difficult owing to lack of neck extension related to cervical spine abnormalities or skull dysplasia. Anatomical variations may be encountered during regional blockade of the brachial plexus.

Consideration of the postoperative management in the context the patient’s existing impairments is crucial. Involvement of physiotherapists and occupational therapists preoperatively will help to predict the support required and any modifications to standard postoperative protocols. Ideally, prolonged use of bulky external supports, such as plaster of Paris or a brace, should be avoided as they can be very limiting for these patients.

## Discussion

This article attempts to provide a multi-disciplinary view on thalidomide upper limb embryopathy, combining the perspectives of scientists, paediatric and adult upper limb surgeons. Understanding how thalidomide affected the embryo is important for several key reasons: it aids identification of survivors who may not be currently recognized as victims of the tragedy (Mansour et al., 2019; Vargesson, 2019); it helps identify new forms of thalidomide that retain clinical benefits (for example in the treatment of leprosy and multiple myeloma) without the risk of inducing birth differences (Vianna et al., 2011); and by understanding the complete mechanism of thalidomide embryopathy it is hoped that similar work will be stimulated for genetic and chromosomal induced syndromes in which the genes or chromosomes have been identified, but the mechanism by which the variation causes the birth differences remains unknown. Ultimately, this may lead to common mechanisms of birth differences being uncovered and the identification of suitable and more appropriate therapeutic strategies. Further collaborative work between surgeons and scientists may yield interesting results. For example, identifying the angiogenic mechanisms of thalidomide when combined with vascular studies in human limbs may shed light on the pathogenesis of conditions like radial dysplasia and hypoplastic thumb.

As our knowledge of the anti-angiogenic action of thalidomide increases, it opens the possibility of using animal models to engineer analogues of thalidomide that retain its clinical benefits without the anti-angiogenic action, thus making a safe form that would not harm the forming embryo or foetus, if taken during pregnancy by mistake. Indeed, many such analogues have been identified and new versions of thalidomide-related analogues have been created that offer neuroprotective actions without causing harm to developing embryos (Beedie et al., 2016; Lecca et al., 2023).

The field of engineered analogues is moving forward rapidly, in part owing to the increased use of thalidomide to treat an increasing range of clinical conditions, including complications of COVID-19 (Vargesson and Stephens, 2021). It is likely that the beneficial actions in the adult, such as stabilizing vessels and preventing new vessel growth to treat conditions like HHT (Lebrin et al., 2010; Peng et al., 2015) will continue to be damaging to exposed embryos but improved understanding of these actions will help determine how thalidomide caused damage to the embryo and provide therapeutic insight into the treatment of such damage after birth.

The clinical experience gained from the early management of these patients as infants should not be underestimated. Although a tragedy, the large number of children who presented with thalidomide-induced congenital differences allowed a great deal of learning, with several landmark articles published in the treatment of congenital hand differences. These articles remain relevant, and the techniques are still being used, in particular the operation of centralization for radial dysplasia (Lamb, 1977). Other knowledge gained from that era included the classification of phocomelia and also the use of prostheses (Lamb, 1971).

Management by clinicians and centres with experience of adult thalidomide patients is necessary to manage age-related symptoms. The use of PROMs questionnaires and other more recent assessment methods highlight not just their current plight but also what may await any adult patient with a congenital hand difference, whether induced by thalidomide or not and should alert medical professionals to their ongoing needs.
